# Prevalence and factors associated with Diabetes retinopathy among type 2 diabetic patients at Northwest Amhara Comprehensive Specialized Hospitals, Northwest Ethiopia 2021

**DOI:** 10.1186/s12886-022-02746-8

**Published:** 2023-01-05

**Authors:** Alebachew Ferede Zegeye, Yemataw Zewdu Temachu, Chilot Kassa Mekonnen

**Affiliations:** 1 Department of Medical Nursing, School of Nursing, University of Gondar, P.O.Box 196, Gondar, Ethiopia; 2 Department of Emergency and Critical Care, School of Nursing, University of Gondar, P.O.Box 196, Gondar, Ethiopia

**Keywords:** Ethiopia, Associated factor, Diabetes mellitus, Diabetic retinopathy

## Abstract

**Introduction:**

The worldwide prevalence of Diabetic Retinopathy was recently estimated to be 34.6%. The prevalence of diabetic retinopathy in developed nations has been thoroughly investigated, and risk factors are well understood. However, there is a shortage of information in the study areas about the prevalence and contributing factors of diabetic retinopathy among type two diabetes patients.

**Objective:**

The aim of this study was to assess the prevalence of diabetic retinopathy and associated factor among type 2 diabetic patients who were on follow up services at northwest Amhara comprehensive specialized hospitals diabetic care units.

**Method:**

An institutional based cross-sectional study was conducted at northwest Amhara comprehensive specialized hospitals from October 15 to November 15, 2021, among 496 diabetes patients. Systematic random sampling technique was used. Data were collected by utilizing a semi-structured questionnaire and a direct Topcon retinal camera inspection. Then data were coded, entered, and exported to SPSS version 23 from EPI-Data version 4.6. All variables with *P*-value < 0.25 in the binary logistic regression analyses were included in the multivariable regression analysis. The degree of association was interpreted by using the adjusted odds ratio with 95% confidence intervals, and the significance level was declared at *P*-value < 0.05. The Hosmer–Lemeshow test was used to check the fitness of the model.

**Result:**

The prevalence of diabetic retinopathy among type two diabetes patients was 36.3%. Sex [AOR = 3.25, 95% CI (1.80, 6.68)], visiting health institution [AOR = 0.027, 95% CI (0.003, 0.253)], educational level [AOR = 4.23, 95% CI (1.09, 16.47)], glycemic control [AOR = 0.099, 95% CI (0.02, 0.49)], hypertension status (AOR = 2.56, 95% CI (1.01, 6.45)] were significantly associated with diabetic retinopathy.

**Conclusion:**

In this study less than half of diabetic patients had diabetic retinopathy. Sex, visiting health institution, educational level, glycemic control, and hypertension status were significantly associated with diabetic retinopathy.

## Background

Diabetes mellitus is a chronic disease of elevated blood glucose levels due to either suboptimal production of insulin by the pancreas or peripheral resistance of the body to insulin [1]. The main causes for DM are defects in insulin secretion, insulin action, or both [2]. It is the leading cause of end-stage renal disease (ESRD), traumatic lower extremity amputations, cardiovascular diseases, and adult blindness [3].

Globally the prevalence of diabetes mellitus in 2014 was 8.5% among the adult population [4]. The prevalence of diabetes has been steadily increasing for the past three decades and is growing most rapidly in low and middle-income countries [5]. Diabetic retinopathy is a known complication of diabetes mellitus and it is characterized by varying degrees of microaneurysm, haemorrhage, hard exudates, cotton-wool spots, venous changes, and new vessel formation involved in the peripheral retina, macula, or both [6]. Globally, the prevalence of diabetic retinopathy among diabetic patients is estimated to be 27.0%, which leads to 0.4 million blindness in the world [7]. Based on a pooled analysis of various hospital-based studies the prevalence of diabetic retinopathy is reported to be 31.6% in Africa [8] and 19.48%in Ethiopia [9]. The International Diabetic Federation estimated that the global prevalence of diabetic retinopathy in 2019 was more than 25% [10].

Globally, visual impairment has decreased, but the number of people who are blind as a result of diabetic retinopathy grew from 0.2 million to 0.4 million [11]. One of the leading global causes of irreversible blindness and the main cause of blindness in adults of working age is diabetic retinopathy. Approximately 80% of people with type 2 diabetes are thought to develop retinopathy [12, 13].

In Ethiopia, the prevalence of diabetic retinopathy at study conducted in Jima, Arbaminch and Debremarkos hospitals was 41.4%, 13%, and 18.5% respectively [5, 14, 15].

In recent years, certain measures have been taken to control diabetic retinopathy. WHO launched the Global Diabetes Compact, a global initiative aiming for sustained improvements in diabetic retinopathy prevention and care, with a particular focus on supporting low- and middle-income countries [16]. Even though, the prevalence of diabetic retinopathy and its associated factors in developed nations has been thoroughly investigated, there is a shortage of information in the study areas about the prevalence and contributing factors of diabetic retinopathy among type two diabetes patients. Therefore, there is a need for more information on the prevalence of diabetic retinopathy in addition to its associated factors in Ethiopia.

### Objective

To assess the prevalence of diabetic retinopathy and associated factors among type 2 diabetic patients attending at Northwest Amhara comprehensive specialized Hospitals, Northwest Ethiopia, 2021.

## Methods

### Study design and period

An institutional-based cross-sectional study was implemented from October 15 to November 15, 2021.

### Study area

The study was conducted from October 15 to November 15, 2021, at Comprehensive Specialized Hospitals in the Northwest Amhara regional state. According to the Amhara National Regional Health Bureau’s Annual Performance Report, the region has 81 hospitals, 858 health centres, and 3560 health posts [17]. Among those 81 hospitals in the region, **t**here are a total of four comprehensive specialized Hospitals in the Northwest Amhara Region such as; Debre Markos, Felege Hiwot, Tibebe Gion, and University of Gondar hospital are found in the Northwest of Amhara. Each comprehensive specialized Hospital serves 3.5–5 million people.

### Source and study population

#### Source population

The source population was all diabetes patients who attend Northwest Amhara comprehensive specialized Hospitals, diabetic clinic.

#### Study population

The study population was all adult diabetes patients who attend Northwest Amhara comprehensive specialized Hospitals, diabetic clinic during the data collection period.

### Inclusion and exclusion criteria

#### Inclusion criteria

Adult diabetes patients who were on diabetic follow up during the study period were included in the study.

#### Exclusion criteria

All diabetes patients with incomplete medical chart recorded were excluded from the study.

### Sample size and sampling procedure

Sample size (N) was calculated using single population proportion formula. Considering the following assumption: standard normal distribution with confidence interval (CI) of 95% (Z = 1.95), tolerable margin of error (d = 0.04), and anticipated proportion of diabetic retinopathy 14.8% (p) taken from a study conducted in Arbaminch

$$\begin{array}{c}\mathrm n=\;{({\mathrm Z}_{{\mathrm\alpha/2}_{}})}^2\;\mathrm p\;(1-\mathrm p)/\mathrm d^2\\\mathrm n=\;{(1.96)}^2.\;0.148(1-0.148)/\;{(0.04)}^2\\\mathrm n=\;303\end{array}$$

By considering 1.5 design effects the final sample size was calculated as:

$$\mathrm{Design}\;\mathrm{effect}\;=\;303\;\times\;1.5=\;455$$

Adding 10% none response rate and the final sample size were 500.

- Where n = minimum sample size required to the study
- d = margin of error
- p = prevalence of diabetic retinopathy
- Z_α/2_ = value of standard normal distribution

### Sampling procedure

To select study subject systematic random sampling was used from those who full fill the inclusion criteria. The patient’s follow-up register in the Diabetic Clinic was used as a sampling frame.

### Variables

#### Dependent variable

- ✓ diabetic retinopathy

#### Independent variable

- ➢ Sociodemographic variables:
-
  - ✓ Age of the patient Sex (male, female) Education level (no education, primary, secondary, diploma and above), Place of Residency (urban, rural) Income of the patient, Occupation
- ➢ Clinical factors
-
  - ✓ Hypertension, Blood glucose HbA1C, Chronic kidney disease, BMI, Duration of illness, Treatment modality
- ➢ Diabetic care
-
  - ✓  Number of visit, Attending health education

### Operational definition

Diabetic retinopathy: on retinal camera examination the presence of micro aneurysms, hemorrhages, exudation, cotton wool spot, and/or new vessels [18]

Background diabetic retinopathy: on retinal camera examination the presence of micro aneurysms, hemorrhages, exudation, and/or cotton wool spot [18]

Maculopathy: on retinal camera examination the presence of exudates within 1 disc diameter (DD) of the fovea, circinate/tracking within 2DD of the fovea [19]

Pre-proliferative DR:—on retinal camera examination the presence of 5 or more cotton wool spots, large blot hemorrhages, intra-retinal micro vascular abnormalities (IRMAs), and/or venous abnormalities [32].

Proliferative DR:—on retinal camera examination the presence of new vessel elsewhere, new vessel on disc and/or pre-retinal/vitreous hemorrhage [19]

### Data collection instruments and procedures

Data was collected using semi-structured questionnaire chart review and direct Topcon retinal camera examination. The questionnaire was adapted from different literature with modification. The investigators have developed the questionnaire for face to face interviews with diabetic patients. The questionnaire has four-part, part 1 socio-demographic variable, part 2 diabetic cares, part 3 treatment modality, and part 4 clinical factors. Data were collected by 4 optometric nurses (one optometric nurse for one hospital) and one supervisor who are trained about diabetic retinopathy screening and who are working in each study area during the study period. Those patients who attend diabetic clinic and fulfill the inclusion criteria have undergo Topcon retinal camera examination following pupil dilation. The laboratory investigation like, fasting blood glucose, glaciated hemoglobin value were collected from the patient’s document.

### Data processing and analysis

After data collection, the collected data were cleaned and checked for completeness. Data were entered by using Epi data version 4.6, after being coded and analyzed using SPSS version 23. Descriptive statistics was used in the analysis of medians, frequencies, and percentages for all variables. Data were presented in tables and charts. The association between dependent and independent variables was assessed by using a binary logistic regression analysis model. Adjusted odds ratio (AOR) was used to estimate the strength of association. All variables associated with diabetic retinopathy with a *p*-value less than 0.25 in the bivariable analysis, was further analyzed using multivariable analyses to control potential confounding factors. Variables with a *p*-value less than 0.05 were declared to be associated with Diabetic retinopathy.

### Data quality control

A pre-test was done on 5% of the total sample size to make sure whether the questionnaire is appropriate and to ensure its validity in the study population before the actual data collection period. After pretest, training was given to all data collectors and supervisors on the purpose of the study, how to get informed consent and the technique of selecting the study participants from each diabetic clinics. Supervision was conducted by the supervisors and principal investigators. All questionnaires were translated into local languages (Amharic) before data collection. Consistency was checked by a back-translation by a language expert both in English and in local languages. At the end of each data collection day, the supervisors were checked for completeness or fulfillment of the questionnaires and the quality of the recorded information.

## Result

### Socio-demographic characteristics

A total of 496 participants were included in this study with a response rate of 99%. Diabetic retinopathy was observed among 180 diabetic patients. With regard to gender distribution more than half (58.1%) of were male. Considering to educational status more than one-third were (36.7%) of them were having college and above education level. Around 188(37.9%) of study populations were employs meaning they have paying job either from governmental or nongovernmental organizations. Regarding monthly income half of the respondents have 1000–3000 birr with median 2000 & IQR ± 2000 birr (Table 1).

**Table 1:** Socio-demographic characteristics of patients attending at diabetic clinics at Northwest Amhara comprehensive specialized Hospitals, 2021 (*n* = 496)

s.n	Variables	Frequency (n)	Percent (%)	Remark
1	**Age in years**			
	18–30	97	19.6%	Median = 52 IQR = ± 23
	31–43	86	17.4%	
	44–56	149	30%	
	> 57	164	33%	
2	**Sex**			
	Male	288	58.1%	
	Female	208	41.9%	
3	**Marital Status**			
	Married	391	78.8%	
	Single	79	15.9%	
	Divorced & widowed	26	5.2%	
4	**Educational Level**			
	No education	75	15.1%	
	Primary education	97	19.6%	
	Secondary education	142	28.6%	
	Diploma and above	182	36.7%	
5	**Occupation**			
	Student	21	4.2%	
	Self employed	159	32.1%	
	Employee	188	37.9%	
	Unemployed	63	12.7%	
	House wife	40	8.1%	
	Other	25	5.0%	
6	**Religion**			
	Catholic	27	5.4%	
	Muslim	54	10.9%	
	Orthodox	396	79.8%	
	Protestant	19	3.8%	
7	**Residence**			
	Urban	342	69.0%	
	Rural	154	31.0%	
8	**Ethnicity**			
	Amhara	340	68.5%	
	Oromo	94	19.0%	
	Tigray	43	8.7%	
	Other^a^	19	3.8%	
9	**Income in ETB**			
	< 1000	129	26.0%	Median & IQR = 2000 Birr
	1000–3000	248	50.0%	
	3001–5000	83	16.7%	
	> 5000	36	7.3%	

*NB*: ^a^Others include farmer and daily labor, Somalia, Southern Nation and Nationality & Afar

### Clinical factors, treatment modality and diabetic care of the patient

In this study the number of hypertensive patients was 168(33.9%). More than half, 347(70%) of the patients whose glycemic level were less than 7 and the other 149(30%) were patient whose glycemic level were greater than 7. About one third, 131(26.4%) of the patient had a body mass index (BMI) of 18.5–24.9 kg/m2. Among those 496 patients in the study 95(19.2%) of them used insulin and 237(47.8%) of them used oral antiglycemic and the remaining 164(33%) used both insulin and oral antiglycemic agents. About 248(50.1%) of the patients visit health institution for their diabetic case every six month. Nearly half, 209(42.1%) of the patient attend the education about diabetic which is given in the hospital (Table 2).

**Table 2:** Clinical, treatment modality and diabetic care factors among type 2 diabetic patients attending at diabetic clinic at Northwest Amhara comprehensive specialized Hospitals, 2021(*n* = 496)

Variables	Frequency (N)	Percent (%)
Hypertension		
Yes	168	33.9%
No	328	66.1%
CKD		
Yes	63	12.8%
No	433	87.2%
HbA1C		
≥ 7	347	70.%
< 7	149	30%
BMI		
18.5–24.9	131	26.4%
25–30	279	56.3%
> 30	86	17.3%
Treatment Modality		
Insulin	95	19.2%
Oral	237	47.8%
Both	164	33%
Number of visit		
Every 1 month	107	21.6%
Every 3 month	141	28.3%
Every 6 month	248	50.1%
Attending health education		
Yes	209	42.1%
No	287	57.9%

### Prevalence of diabetic retinopathy among type 2 DM patients

**Fig. 1:**
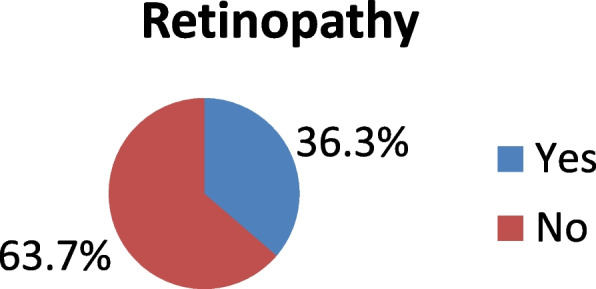
Prevalence of diabetic retinopathy and associated factors among type 2 diabetic patients attending at Northwest Amhara comprehensive specialized Hospitals, Northwest Ethiopia, 2021

The result of this study showed that 36.3% with 95% CI (29.8, 47.6) of study participants had had diabetic retinopathy and the remaining 313(63.7%) did not have diabetic retinopathy. Therefore, as shown in Fig. 1 below the prevalence of retinopathy among type 2 diabetic patient was 36.3%

### Associated factors of diabetic related retinopathy

Sex, visiting health institutions every month for diabetic care, an educational level which is a diploma and above, glycemic control less than 7, and having hypertension were found to have a significant association with diabetic retinopathy among type two diabetic patients.

For males, the odds of diabetic retinopathy were 3.25 times that of females [AOR = 3.25, 95% CI (1.80, 6.69)]. Patients who visit health institutions for their diabetic case every month had about 97% [AOR = 0.027, 95% CI (0.003, 0.253)] less chance of diabetic retinopathy than those patients who visit health institutions for their diabetic case every six months. The odds of no education were 4.23 times compared to a diploma and above [AOR = 4.23 95% CI (1.09, 16.47)]. It was also observed that patients with glycemic control less than 7 had about 9.9% less chance [AOR = 0.099, 95% CI(0.02, 0.49)] to diabetic retinopathy compared with patients with glycemic control greater than 7. Hypertensive patients had about 2.56 times the chance of diabetic retinopathy than non-hypertensive patients [AOR = 2.56, 95% CI (1.01, 6.45)]. The other covariates age, residence, occupation and BMI does not have a significant effect on Diabetic retinopathy (Table 3).

**Table 3:** Factors associated with diabetic retinopathy among type two diabetic patients attending diabetic clinic at Northwest Amhara comprehensive specialized Hospitals, 2021(*n* = 496)

Variables	**Retinopathy**		**OR (95% CI)**	
	Yes	No	**COR 95% CI**	**AOR 95% CI**
Sex				
Male	135	113	1.90(1.08, 3.49)	**3.25(1.80, 6.69)**
Female	97	151	1	1
Educational Level				
No education	22	53	7.14(3.18, 16.02)	**4.23(1.09, 16.47)**
Primary education	28	69	6.98(3.22, 15.14)	6.20(0.83, 21.09)
Secondary education	16	126	2.18(0.96, 4.97)	2.52(0.71, 8.89)
Diploma and above	10	172	1	1
Occupation				
Student	5	16	0.31(0.10, 0.97)	1.11(0.14, 9.08)
Self-employee	14	145	0.11(0.03, 0.38)	1.51(0.24, 9.40)
Employee	6	182	3.10(1.01, 9.49)	2.21(0.34, 5.42)
Unemployed	31	32	1.72(0.52, 5.70)	0.80(0.17, 1.35)
House-wife	14	26	1.01(0.26, 3.94)	0.03(0.44, 2.16)
Other	6	19	1	1
Income				
< 1000	50	79	3.17(1.23, 8.15)	1.03(0.36, 2.51)
1000–3000	14	234	0.30(0.11, 0.84)	0.06(0.07, 4.08)
3001–5000	6	77	0.39(0.12, 1.30)	0.21(0.03, 1.06)
> 5000	6	30	1	1
HbA1C				
≥ 7	19	234	0.27(0.107, 0.60)	**0.099(0.02, 0.49)**
< 7	57	186	1	1
Hypertension				
Yes	65	262	3.56(1.07, 5.59)	**2.56(1.01, 6.45)**
No	11	158	1	1
BMI				
18.5–24.9 kg/m^2^	42	373	1	1
25 – 30 kg/m^2^	20	34	0.48(0.22, 1.05)	1.10(0.10, 2.42)
> 30 kg/m^2^	14	13	1.12(1.28, 4.23)	1.01(0.11, 6.87)
Number of visit				
Every 1 month	13	19	0.30(0.14, 0.67)	**0.027(0.003, 0.253)**
Every 3 month	22	302	0.71(0.37, 1.36)	0.640(0.125, 3.32)
Every 6 month	41	99	1	1

*NB*:- variables statistically significant in multivariable logistic regression with *p*-value

< 0.05*, COR stands for crude odd ratio. AOR stands for adjusted odd ratio, *1 stands for reference

## Discussion

Diabetic retinopathy (DR) is a well-known micro vascular complication of diabetes mellitus (DM) and it is one of major global health concern, which places a huge burden on the health care system [8].

The result of this study showed that 36.3% with 95% CI (29.8, 47.6) of study participants had diabetic retinopathy. This finding is in line with a study conducted in Armenia 36.2% [20]. Even though there is a difference in socioeconomic status and level of health sector development, the possible reason for the similarity between the current study and the study in Armenia might be using a similar study population (diabetic patients), study unit and study design.

This finding is higher than a study conducted in Arbaminch which is 13% [21], and in China 8.1% [22]. However, this finding is lower than a study conducted in Jimma university Hospital 41.4% [23], Kenya 41% [24], and Babol teaching hospitals, Iran 64.1% [25]. The possible reason for this difference might be due to differences in methodology, sample size, time variation in the study period and different health-seeking behaviour among the study participant.

In the binary logistic regression model, five variables are found to be predictive factors for diabetic retinopathy. These variables are sex, number of visits, glycemic control, educational level, and hypertension status.

The odds of diabetic retinopathy for male were 3.25 times that of females [AOR = 3.25, 95% CI (1.80, 6.69)]. This study was consistent with the study conducted in Pakistan and wales [26, 27]. But a study conducted in Embu Provincial General Hospital, Central Kenya revealed that there was no discernible sex difference in the prevalence of any diabetic retinopathy [28]. The possible explanation might be Neuroretinal function is more abnormal in males than in females for adults with type 2 diabetes. These results suggest that, relative to males, females may have some protection from, or resistance to, neurodegenerative changes that precede the development of background retinopathy in type 2 diabetes [29].

The odds of diabetic retinopathy for diabetic patients who visit health institutions for their diabetic case every month had about 2.7% [AOR = 0.025, 95% CI (0.003, 0.253)] less chance diabetic retinopathy than those patients who visit health institutions for their diabetic case every six months. A similar finding was reported in a study conducted in Ghana [30].This may be due to, most diabetic patients in this study being appointed every 6 months and more. This contributes to poor glycemic control. Moreover, visits to the clinic were limited to the prescription of medications.

The odds of diabetic retinopathy among diabetic patients who had no education was 4.23 times [AOR = 4.23 95% CI (1.09, 16.47)] compared to diabetic patients who had a diploma and above. This study was consistent with the study conducted in Japan [31]. The possible explanation might be Individuals with lower educational levels have limited income, poor occupational opportunities, and reduced access to healthcare services and information. Therefore patients with the lowest socioeconomic status and household income have been shown to have the highest prevalence of retinopathy [32].

The odds of diabetic retinopathy among diabetic patients with glycemic control (HbA1c less than 7) had 9.9% [AOR = 0.099, 95% CI (0.02, 0.49)] less risky for diabetic retinopathy when compared to patients with glycemic control (HbA1c greater than 7). This finding is in line with a study done in Tanzania and Australia [33, 34]. The possible explanation might be individuals with poor glycemic control had a chance of nearly 4 times the risk for diabetic retinopathy. Glycemic control remains an important factor in the presence and progression of diabetic retinopathy. HbA1c seems to be an indicator which can demonstrate exactly the degree of glycemic control, while sudden variations of blood glucose may play an important role in diabetic retinopathy; therefore, glycemic control may be useful to predict DM complications, such as diabetic retinopathy.

The odds of diabetic retinopathy among diabetic patients with hypertension had about 2.56 times [AOR = 2.56, 95% CI (1.01, 6.45)] chance of diabetic retinopathy than non-hypertensive patients. This finding is similar to the study conducted in Khartoum, Sudan and Arbaminch, Ethiopia [5, 35]. A possible explanation might be Diabetic retinopathy prevalence is related to blood pressure levels [36]. It has been observed that the prevalence of hypertension is higher in diabetic subjects than in the general population and it also plays a major role in the progression of diabetic retinopathy. The possible mechanisms by which hypertension affects diabetic retinopathy are hemodynamic (impaired autoregulation and hypoperfusion) and secondly through Vascular Endothelial Growth Factor, as it has been observed that hypertension independent of hyperglycemia up-regulates the Vascular Endothelial Growth Factor expression in retinal endothelial cells and ocular fluids [37].

### Strength and limitations

This study is the first to apply multicenter approach in the Northwest Amhara region to assess prevalence and factors associated with Diabetes retinopathy among type 2 diabetic patients. However, the study adopted a cross-sectional study design and comprised a relatively smaller number of participants.

## Conclusion

This study revealed that the prevalence of diabetic retinopathy was high (36.3%). Sex, number of visits, educational level, glycemic control and hypertension were found to have significantly associated with diabetic retinopathy.

This study result will help as a guideline for decision-makers and program planners at the time to design and implement of intervention. Moreover, the result of this study could be used as a literature for future researchers and be a clue for further studies to be done on the prevalence of retinopathy among diabetes type 2 patients.

## Data Availability

Data will be available upon request from the corresponding author.

## Abbreviations

AOR: Adjusted odds ratio
BMI: Body mass index
CKD: Chronic kidney disease
COR: Crude odds ratio
DD: Disc diameter
DM: Diabetes mellitus
DR: Diabetic retinopathy
ESRD: End stage renal disease
ETDRS: Early treatment diabetic retinopathy study
IQR: Inter quartile range
NPDR: Non-proliferative diabetic retinopathy
OR: Odds ratio
USA: United States of America
